# Neural Mechanisms of Cognitive Dissonance (Revised): An EEG Study

**DOI:** 10.1523/JNEUROSCI.3209-16.2017

**Published:** 2017-05-17

**Authors:** Marco Colosio, Anna Shestakova, Vadim V. Nikulin, Evgeny Blagovechtchenski, Vasily Klucharev

**Affiliations:** ^1^Center for Cognition and Decision Making, National Research University Higher School of Economics, 109316, Moscow, Russian Federation,; ^2^Department of Neurology, Max Planck Institute for Human Cognitive and Brain Sciences, 04103 Leipzig, Germany, and; ^3^Laboratory of Neuroscience and Molecular Pharmacology, Institute of Translational Biomedicine, Saint Petersburg State University, Saint Petersburg, Russian Federation 199034

**Keywords:** cognitive dissonance, error-related negativity, long-range temporal correlation, resting state, spread of alternatives

## Abstract

Cognitive dissonance theory suggests that our preferences are modulated by the mere act of choosing. A choice between two similarly valued alternatives creates psychological tension (cognitive dissonance) that is reduced by a postdecisional reevaluation of the alternatives. We measured EEG of human subjects during rest and free-choice paradigm. Our study demonstrates that choices associated with stronger cognitive dissonance trigger a larger negative frontocentral evoked response similar to error-related negativity, which has in turn been implicated in general performance monitoring. Furthermore, the amplitude of the evoked response is correlated with the reevaluation of the alternatives. We also found a link between individual neural dynamics (long-range temporal correlations) of the frontocentral cortices during rest and follow-up neural and behavioral effects of cognitive dissonance. Individuals with stronger resting-state long-range temporal correlations demonstrated a greater postdecisional reevaluation of the alternatives and larger evoked brain responses associated with stronger cognitive dissonance. Thus, our results suggest that cognitive dissonance is reflected in both resting-state and choice-related activity of the prefrontal cortex as part of the general performance-monitoring circuitry.

**SIGNIFICANCE STATEMENT** Contrary to traditional decision theory, behavioral studies repeatedly demonstrate that our preferences are modulated by the mere act of choosing. Difficult choices generate psychological (cognitive) dissonance, which is reduced by the postdecisional devaluation of unchosen options. We found that decisions associated with a higher level of cognitive dissonance elicited a stronger negative frontocentral deflection that peaked ∼60 ms after the response. This activity shares similar spatial and temporal features as error-related negativity, the electrophysiological correlate of performance monitoring. Furthermore, the frontocentral resting-state activity predicted the individual magnitude of preference change and the strength of cognitive dissonance-related neural activity.

## Introduction

Normative decision theory suggests that our actions reflect our preferences, whereas the influential theory of “cognitive dissonance” (Festinger, 1957) postulates that our actions shape our preferences. Numerous studies have shown that, when a person must select between two equally attractive items, the act of choosing one item will induce a preference change (for review, see Izuma et al., 2010). The theory of cognitive dissonance suggests that such difficult choices could cause psychological discomfort (cognitive dissonance), which forces people to engage mechanisms of conflict reduction and preference change (for review, see Harmon-Jones and Harmon-Jones, 2008). According to the action-based model of cognitive dissonance, activity in the posterior medial frontal cortex (pMFC) underlies detection of cognitive conflicts and the reduction of cognitive dissonance (Carter et al., 1998; Amodio et al., 2004; Izuma et al., 2010). Nevertheless, the neurocomputational foundation of cognitive dissonance remains unclear. Here, we further studied the role of the pMFC in cognitive dissonance and preference change.

Interestingly, the pMFC has also been implicated in the generation of a “reward prediction error” signal when the outcome of an action differs from the expected one (Holroyd and Coles, 2002; Nieuwenhuis et al., 2004; Cohen and Ranganath, 2007; Rushworth et al., 2007; see also Botvinick, 2007). This signal presumably guides future action selection by updating predictions of action values (Niv, 2009). Involvement of the pMFC in cognitive dissonance and general performance monitoring may suggest that cognitive dissonance, general action monitoring, and reinforcement learning may share neural mechanisms. Thus, we explored whether difficult choice-induced preference changes can be driven by a neural mechanism similar to the general mechanism of performance monitoring and behavioral adjustment.

We further hypothesized that choice-induced preference changes depend on resting-state pMFC neuronal dynamics. Recent neuroimaging studies have shown that performance in the motor task (Smit et al., 2013) and perceptual tasks (Palva et al., 2013) may be related to the long-range temporal correlations (LRTCs) of neuronal oscillations recorded at rest conditions. Importantly, LRTCs indicate the presence of a scale-free structure of neuronal activation on multiple time scales that is important for optimal neuronal processing in the human brain (Linkenkaer-Hansen et al., 2001; Hardstone et al., 2012; Palva et al., 2013). Here we suggest that the magnitude of cognitive dissonance can be predicted by the resting-state neuronal dynamics recorded with an EEG before a cognitive dissonance-inducing task. Specifically, we hypothesized that LRTC and the amplitude of frontal alpha oscillations would correlate with the behavioral and electrophysiological indices of dissonance-induced preference change.

To clarify the mechanism of cognitive dissonance, we used an electrophysiological signature of behavioral error monitoring: the error-related negativity (ERN) component, a negative frontocentral deflection in the event-related potential (ERP). ERN is generated in the pMFC (Holroyd and Coles, 2002; Holroyd et al., 2004; Ridderinkhof et al., 2004; Debener et al., 2005) and has been associated with processing errors (Holroyd et al., 2003), monitoring of action outcomes (Luu et al., 2004), and behavioral adjustments (Gehring et al., 2011).

The decrease in ratings for rejected items, also known as Spread of Alternatives (SoA), has been repeatedly demonstrated under the “free-choice paradigm” (Brehm, 1956; Gerard and White, 1983; Shultz et al., 1999; Lieberman et al., 2001; Coppin et al., 2010). Here, we tested the hypothesis that choice-induced preference changes are associated with a response-locked negative ERP similar to ERN: We expected that a larger ERN-like activity would be generated during difficult decisions than easy decisions. We recorded ERPs during both the free-choice paradigm and the Eriksen Flanker task (Eriksen and Eriksen, 1974), the latter of which can be used as an ERN “functional localizer” task. Overall, our approach allowed us to investigate similarity between neural mechanisms involved in choice-induced preference changes and more general reinforcement-learning mechanisms.

## Materials and Methods

### Participants

Forty-five right-handed, healthy participants (20 males, mean age 22.17 ± 2.68 years) were recruited and provided a small amount of compensation (equivalent to $12–15 US dollars). Participants were instructed to fast for at least 3 h before the study.

All 45 participants underwent a version of the free-choice paradigm (Izuma et al., 2010). For 24 participants (11 of whom were males), we also recorded classical ERN during the Eriksen Flanker task (for details, see below). Three participants were excluded from the analysis of the free-choice paradigm due to clear instructional misunderstanding. Thus, 42 and 24 subjects participated in the analysis of the free-choice paradigm and Eriksen Flanker task, respectively.

All participants had normal or corrected-to-normal vision and received no regular medications. None of the subjects had a history of neurological or psychiatric illness. The study protocol was approved by the local ethics committee.

### Experimental tasks

#### Free-choice paradigm

##### Stimuli.

A set of 446 digital color photos of snack foods on a white background (chocolate, chips, small fruits or vegetables, cheese) were used as stimuli. The items were selected from a larger dataset during a prestudy to incorporate the most familiar food items available in the local market. The price of each item was below $8 US dollars (500 rubles). The photos were projected onto a screen, with a visual angle of 4.772° vertically and 7.62° horizontally.

##### Procedure.

**Figure 1.**
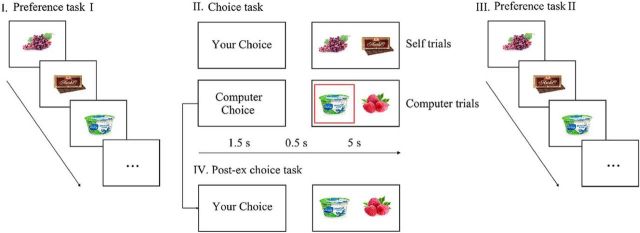
Free choice paradigm. I. During Preference task I, participants rated food items presented for 3 s on the screen. II. Next, during Choice task in Self-trials, subjects freely selected one of two food items (Self-difficult trials evoked strong cognitive dissonance, Self-easy trials evoked weak cognitive dissonance), whereas in Computer trials, subjects had to select the item that has been selected by the computational algorithm (highlighted by a red square). III. In Preference task II, participants rated the same food items again. Participants were reminded about their choices (if any) during the Choice task (e.g., “You rejected it”). IV. Finally during control Post-ex Choice task, participants chose items from pairs that had been presented during Computer trials.

The basic free-choice paradigm (Fig. 1) consisted of three main parts: I, Preference task I; II, Choice task; and III, Preference task II.

During Preference task I, participants rated a set of 446 food items using an 8-point Likert scale (1 = “I don't like it at all” to 8 = “I like it a lot”). Each item appeared at the center of the screen for 3 s. During the Choice task, two food items were presented on the screen at the same time (up to 210 pairs altogether). In Self-trials, participants were instructed to choose one food item that they preferred. To increase their motivation, participants were informed that they would receive one of the selected food items along with monetary compensation. Unknown to participants, the pairs were created using a computational algorithm based of participants' ratings during Preference task I: one-half of the pairs included two highly preferred food items (rated between 6 and 8; these trials were defined as Self-difficult trials evoking high cognitive dissonance), and the remaining one-half included one highly preferred food item and one unpreferred item (rated <3; these trials were defined as Self-easy trials, evoking low cognitive dissonance). In a control, Computer trials, participants were instructed to press the button corresponding to the food item randomly chosen by the computer (highlighted by a red square). Importantly, in Computer trials, items were programmed and selected with the same criteria used for Self-difficult trials. Overall, each food item appeared in only one pair. At the beginning of each trial, participants were informed about the trial type (Self-trial or Computer trial). Participants had 5 s to make their choice or press the keyboard button corresponding to the computer's choice. In case of no answer, a written message, “Please, respond faster,” appeared at the center of the screen.

During Preference task II, participants had to rate the same set of food items again. Unlike Preference task I, an additional text indicated either the participant's or the computer's decision during the Choice task (e.g., “you chose it,” “you rejected it,” or “computer chose it,” “computer rejected it”). In line with previous studies (e.g., Izuma et al., 2010; Izuma and Murayama, 2013), each food item during Preference task II was presented with information about whether the participant (or the computer) had chosen or rejected the item (e.g., “You rejected this item”). This information could potentially strengthen the effect of cognitive dissonance by highlighting the discrepancy between the attitude and past behavior. Thus, the preference change found in the present study could be inflated due to this aspect of the experimental design. A previous study showed that the reminder cue affected SoA, but the SoA effect was still significant for the “nonreminded” control group (see Salti et al., 2014, who formally test the effect of this explicit feedback). Importantly, the aim of the present study is not to test cognitive dissonance during Preference task II (when this reminding information was presented), but to test its neural signatures earlier, during the Choice task.

Finally, participants attended an additional control condition, a Post-ex Choice task, introduced to address the methodological issue raised by Chen and Risen (2010). They argued that the standard free-choice paradigm could demonstrate a preference change in the absence of cognitive dissonance. Importantly, even if two items are rated equally during the Preference task I, it does not necessarily mean that real preferences for these two items are the same. Thus, a real preference for a chosen item during the Choice task is likely to be higher than a real preference for a rejected good. Then, when participants are asked to rate the same items again, it is more likely that their rating of a chosen item will increase and their rating of a rejected item will decrease on average. Therefore, Chen and Risen (2010) designed a control condition in which participants made a choice after they had made two preference ratings (rate-rate-choose condition, Post-ex Choice task). Items were categorized as “chosen” or “rejected” according to the choices at the end of the experiment, during the Post-ex Choice task. Importantly, in this condition, changes between the two preference tasks cannot be induced by choices (cognitive dissonance). Chen and Risen (2010) indeed found a significant preference change, even in the rate-rate-choose condition, demonstrating that the preference change measured in a typical free-choice paradigm can occur in the absence of cognitive dissonance. To adjust for this confounding effect, the Post-ex choice (rate-rate-choose) control condition has been widely used (for review, see Izuma and Murayama, 2013). Subjects were instructed to choose from the same pairs of food items that had appeared during the Computer trials of the Choice task conditions. In Computer trials and Post-ex Choice task, items were selected with the same criteria used for Self-difficult trials.

At the end of the experiment, we randomly selected one of the items that participants had selected during Self-difficult trials or Post-ex choice trials as an additional reward for the participants.

### Eriksen Flanker task

At the end of the study, a subset of 24 subjects performed the Eriksen Flanker task. A string of 7 elements appeared on the monitor for 150 ms followed by a black screen (600–1000 ms). Each string consisted of a central element (the target) and 3 flankers. The elements were combined as congruent (<<< or >>>) or incongruent (<>< or ><>) stimuli. Participants were instructed to react as quickly and accurately as possible by pressing the correct button according to the orientation (left or right) of the target element, regardless of the orientation of the flankers. If participants responded too late (slower than 800 ms), a message, “you are too late,” prompted them to respond faster. The task consisted of 7 blocks (60 trials per block). Each string type appeared with a probability of 0.25.

### Behavioral measure and analysis

We calculated individual reaction times (RTs) in each condition to relate them to the levels of cognitive conflict. We assumed that a longer RT is associated with a higher level of conflict. To assess the effect of cognitive dissonance on behavioral preference changes, or SoA, we calculated preference change by subtracting the average rating made during Preference task II − from the average rating made during Preference task I, separately for the selected and rejected items and the four experimental conditions (Self-difficult trials, Self-easy trials, Computer choice, and Post-ex choice). A positive preference change indicated an increased postdecisional preference for the food item (more liking), whereas a negative preference change suggested a decreased postdecisional preference for the food item (less liking). SoA (postdecisional preference change) analysis was performed by entering both accepted and rejected item ranks (Preference task II − Preference task I) for each of the experimental conditions into paired *t* tests (for a similar analysis, see Izuma et al., 2010) and two-way ANOVA.

### Procedure

#### Resting-state recordings

At the beginning of the study, subjects sat comfortably in a chair for 10 min with their eyes open while a resting-state recording was performed. Subjects were instructed to relax and not move during the recordings.

### ERP recordings

The EEG data were collected for each subject during the whole experiment at the 500 Hz sampling rate in the frequency range 0.2–100 kHz from 60 high-impedance ActiCap active scalp electrodes (Brain Products), which were positioned according to the international 10–20 system. Impedances were kept <10 kΩ. Eye movements were recorded with Ag/AgCl electrodes placed at both lateral canthi and below the left eye. EEG signals were referenced to arithmetically link mastoids. Offline, the EEG was bandpass filtered in the 0.1–30 Hz frequency range. The data were inspected for artifacts (amplitudes exceeding ±100 μV), and <10% of all trials in each condition and with each participant were rejected.

### Data analysis

#### Analysis of the resting-state recordings

In the present study, we focused on the analysis of alpha oscillations in the resting state for two reasons. First, alpha oscillations have been shown to be involved in many cognitive operations, including memory, attention, and decision making (Klimesch, 1999, 2012; Jensen et al., 2002; Cohen et al., 2009). Second, alpha oscillations have a large signal-to-noise ratio, which facilitates extraction of their amplitude without it being strongly affected by muscle activity (Palva et al., 2005; Palva and Palva, 2007; Klimesch, 2012; Frey et al., 2015). For the extraction of the instantaneous amplitude, we used an analytic signal concept based on the Hilbert transform. Following a previously established practice, we extracted alpha oscillations in low 8–10 Hz and high 10–12 Hz frequency bands as well as in a broad 8–13 Hz range. Mean amplitude was calculated as the average of the instantaneous amplitude over 10 min of rest recordings.

LRTCs of alpha oscillations were estimated with detrended fluctuation analysis (DFA) (Peng et al., 1995; Kantelhardt et al., 2001). LRTCs are a relatively new measure of the intrinsic functional state of a cortical region. Importantly, LRTCs indicate the presence of temporal autocorrelations within the measured cortical area (i.e., LRTCs describe the temporal development of the neuronal activity). Thus, LRTCs do not indicate the connectivity of the brain region but rather its functional state. LRTCs are characterized by a slow (power-law) decay of autocorrelation, which in turn indicates that past neuronal events might affect the activity of the remote upcoming neuronal activity. Such neuronal dynamics were shown to reflect a delicate balance between excitation and inhibition (Poil et al., 2012), thus relating to the performance in a given experimental task (Palva et al., 2013; Smit et al., 2013; Samek et al., 2016).

Algorithmically, DFA captures fluctuations of the signal at different time scales, and the slope of the corresponding line is called a scaling exponent. For random signals, such as white noise, the scaling exponent is 0.5. However, if there are persistent LRTCs, the exponent will lie in the 0.5–1 range. Higher scaling exponents correspond to more pronounced LRTCs.

For DFA, we used 30 time windows, from 5 to 50 s, distributed equidistantly on a logarithmic scale. Further technical details on the application of DFA for the estimation of LRTCs in EEG/MEG signals can be found in Hardstone et al. (2012). We then correlated scaling exponents and the amplitude of the oscillations with the SoA effect in the Self-easy and Self-difficult trials, as well as with the amplitude of evoked responses in the free-choice paradigm. For the behavioral data, we used a region-of-interest (ROI) approach and averaged LRTC scaling exponents in the electrodes belonging to the frontocentral area (F1, F2, F3, F4, Fz, FCz) where ERN was most pronounced. Consequently, we correlated this ROI scaling exponent with the behavioral data. Scaling exponents and amplitudes from this ROI were correlated with the SoA. For the evoked responses, we selected amplitudes from the FCz electrode and then correlated them with the scaling exponents and amplitude of alpha oscillations obtained from all electrodes. In this case, because we had calculations of multiple correlations, we applied permutation tests based on cluster statistics (Maris and Oostenveld, 2007). All analytical steps were performed with scripts implemented in MATLAB (The MathWorks).

### Analysis of ERP

We analyzed response-locked activity in both tasks. In the free-choice paradigm, the response-locked ERPs elicited during Self-difficult trials and Self-easy trials were subjected to an analysis of the effect of cognitive dissonance. Importantly, as all ERPs were response-locked to the motor responses, the difference in RTs was controlled for between Self-difficult and Self-easy trials. To examine ERN, in the Eriksen Flanker task, we calculated the difference between the average response-locked ERPs in trials with both incorrect responses and correct responses.

Additionally, a paired *t* test was performed for the FCz electrode based on the individually averaged ERP responses at the latency window between 0 and 90 ms from the response onset and a 35 ms integration window. A significance of the differences between the conditions was assessed with permutation tests based on cluster statistics (Maris and Oostenveld, 2007). We compared response-locked ERPs in Self-difficult trials to response-locked ERPs in Self-easy trials in the free-choice paradigm. We also compared response-locked ERPs in incorrect responses to response-locked ERPs in correct responses in the Eriksen Flanker task.

### Source localization analysis

We used the low-resolution brain electromagnetic tomography (LORETA) method, implemented in the Brain Vision Analyzer (Brain Products) to identify the neural generator of the ERN and cognitive dissonance-related ERPs. LORETA estimates the current source density distribution in the brain, which contributes to the electrical scalp field (Pascual-Marqui et al., 1994; Pascual-Leone et al., 1999). LORETA computes the smoothest of all possible source configurations throughout the brain volume by minimizing the total squared Laplacian of source strengths (Pascual-Marqui et al., 1994; Pascual-Marqui, 2002; Schneider et al., 2009). Here, we used LORETA to identify the neural generator of the ERN and cognitive dissonance-related ERPs recorded in the Eriksen Flanker task and the free-choice paradigm.

## Results

### Behavioral correlates of choice difficulty

The analysis of RTs showed that Self-difficult trials required a longer RT than did Self-easy trials (*t*_(41)_ = 5.997, *p* < 0.001) and Post-ex choice trials (*t*_(41)_ = 5.995, *p* < 0.001), confirming further the difference in choice difficulty between difficult choices and control conditions. Mean values of RT were 2035 ± 235, 1739 ± 215, and 1640 ± 139 ms for Self-difficult, Self-easy, and Post-ex choice trials, respectively. Interestingly, participants were slower in Computer trials than in Self-difficult (*t*_(41)_ = 3.597, *p* < 0.001), Self-easy (*t*_(41)_ = 4.765 *p* < 0.001), and Post-ex choice (*t*_(41)_ = 5.419, *p* < 0.001) trials. As participants passively observed a computer “selecting” food items, they could implicitly compare the selection to their own preferences for items and prepare motor responses. The significant RT slowdown in Computer trials can be explained by a cognitive conflict occurring when the computer's selections mismatched the participants' preferences (revealed in Post-ex choice trials). Such conflict was indeed reported by participants at the end of the experiment. Thus, a mismatch between the computer's selection and a participant's genuine preference might lead to a longer RT due to an inhibition of the participant's initial choice. Indeed, the RT in Computer trials when selections matched participants' preferences (1809 ± 481 ms) was faster than the RT in Computer trials when selections mismatched participants' preferences (2556 ± 784 ms): *t*_(41)_ = 4.731, *p* = 0.018. The cognitive processes during Computer trials should be further investigated in future studies.

### Behavioral correlates of postdecisional preference change

Because strong cognitive dissonance should occur during difficult decisions (Self-difficult trials and Post-ex choice), we predicted that magnitude of the SoA should be enhanced as a function of choice difficulty. Thus, we expected a larger SoA for Self-difficult and Post-ex choice trials than for Self-easy and Computer trials.

**Figure 2.**
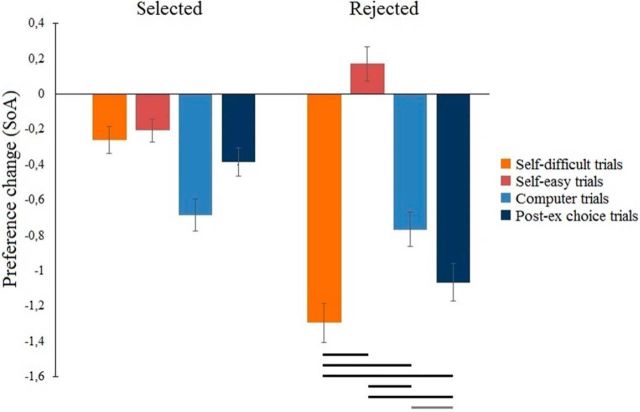
Postdecisional preference changes for selected and rejected items in free choice paradigm. Black lines below the histogram indicate statistically significant postdecisional preference change between Preference task I and Preference task II for rejected items. Black lines indicate *p* < 0.001. Gray line indicates *p* = 0.083. Error bars indicate SEM.

SoA analysis was subjected to a two-way (factor trial type: Self-difficult, Self-easy, Computer trials, and Post-ex choice; factor choice: selected vs rejected) repeated-measures within-subject ANOVA. We found a main effect of trial type (*F*_(3,123)_ = 57.488, *p* < 0.001) and choice (*F*_(1,41)_ = 45.43, *p* < 0.001). The ANOVA also revealed a significant interaction between trial type × choice: *F*_(3,123)_ = 105, *p* < 0.001. As predicted, we observed a significantly larger SoA for rejected items in Self-difficult trials than for rejected items in Self-easy trials (Fig. 2).

In addition, we controlled for a floor effect in Self-easy trials that could prevent a downward valuation of these low-value food items. We reanalyzed the preference changes for food items in Self-easy trials with an initial rating of “3.” Taking into account that the maximum postdecisional preference change in our study was −1.3, these items should be fully susceptible to a downward valuation. The average preference change for the rejected items with an initial rating of “3” (Self-easy trials) was 0.10 ± 0.11. Importantly, the preference change for these items significantly differed from the rejected items in Self-difficult trials: *t*_(41)_ = −9.065, *p* < 0.001. This indicates that the preference changes in Self-easy trials were indeed lower than those in Self-difficult trials, regardless of the floor effect.

We also analyzed the SoA in trials with the highest cognitive dissonance: Self-difficult trials and control Post-ex choice trials. A two-way (factor trial type: Self-difficult, Post-ex choice; factor choice: selected, rejected) repeated-measures within-subject ANOVA showed a significant main effect of choice (*F*_(1,41)_ = 202.92, *p* < 0.001) but not trial type (*F*_(1,41)_ < 1, *p* = 0.770). Crucially, we found significant interaction between trial type × choice (*F*_(1,41)_ = 7.43, *p* = 0.009), indicating a larger SoA for Self-difficult trials than for Post-ex choice trials.

*Post hoc* analyses revealed that participants' preferences for items that were rejected during Self-difficult trials significantly decreased compared with both the rejected items in Self-easy trials (*t*_(41)_ = −11.090, *p* < 0.001) and the selected items in Self-difficult trials (*t*_(41)_ = −12,005, *p* < 0.001). The SoA for items that were rejected during Self-difficult trials was significantly stronger than it was for items rejected or selected in the control Computer trials*: t*_(41)_ = −7.143, *p* < 0.001 and *t*_(41)_ = −7.263, *p* < 0.001), respectively.

The SoA of rejected items in Self-difficult trials approached significance compared with rejected items in another control Post-ex choice condition: *t*_(41)_ = −1.779, *p* = 0.083. However, all the remaining comparisons showed a significant effect (all *p* < 0.001), except for the SoA between accepted and rejected items in Computer trials (*t*_(41)_ = −1.402, *p* = 0.168).

To conclude, our results (Fig. 2) replicate previous SoA findings using the free-choice paradigm (Izuma et al., 2010; Salti et al., 2014). Importantly, the efficiency of the control conditions for the free-choice paradigm is still debated (Coppin et al., 2014), and further investigation is needed. Therefore, the electrophysiological signature of cognitive dissonance during control conditions is marginally discussed in this paper.

Overall, our results showed that (1) participants deevaluated previously rejected items (SoA effect); (2) the SoA effect for rejected items was stronger for more difficult choices associated with stronger cognitive dissonance; and (3) difficult choices are reflected by longer decisional RT.

### ERN in the Eriksen Flanker task

**Figure 3.**
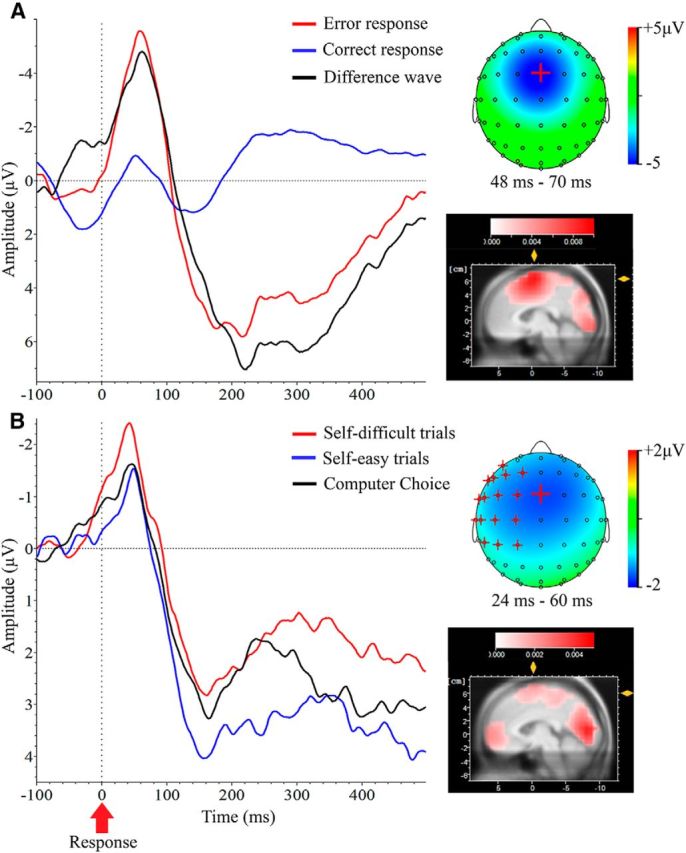
***A***, Left, Grand-averaged ERN (FCz) for correct responses, incorrect responses, and difference wave in the Eriksen Flanker task. Right, Topographical map for difference wave voltage distribution and LORETA solutions (scale range: 0–0.01 μA/mm^2^) for the difference wave within selected time window (±11 ms around the peak). ***B***, Left, Grand-averaged ERPs (FCz) for Self-difficult, Self-easy, and Computer trials in the free-choice paradigm. Right, Topographical map for voltage distribution of the difference wave and LORETA solutions (scale range: 0–0.005 μA/mm^2^) within 36 ms time window. Magenta crosses represent electrodes surviving a 200 random iterations permutation procedure. In both voltage distribution maps, large cross represents FCz electrode. All ERPs are response-locked.

During the control Eriksen Flanker task, error responses were followed by larger frontocentral negativity-ERN compared with correct responses ∼60 ms after the button press. Previous studies have indicated the maximum of ERN at FCz (Yeung et al., 2004). Therefore, we conducted a paired *t* test that showed a significant difference between error responses and correct responses at FCz: *t*_(20)_ = −5.57, *p* = 0.001, *d* = 1.2. The ERN′s time course and topographical map are illustrated in Figure 3*A*.

To localize the generator of the ERN, we applied LORETA transformation to the evoked activity within a preselected time window corresponding to ±10 ms around the grand-averaged maximum peak for difference waves (error responses − correct responses).

As expected, LORETA analysis highlighted a prominent activation of the pMFC, with the strongest activity in the BA6 (for *x*, *y*, *z* and BA coordinates, see Table 1); this finding is similar to previous studies that investigated the ERN generator in the Eriksen Flanker task (Herrmann et al., 2004).

**Table 1.** LORETA solutions: localization of the CD/error-related ERPs at the pMFC in free-choice paradigm and Eriksen Flanker task

Condition	*x* (mm)	*y* (mm)	*z* (mm)	Brodmann area	Task
Self-difficult	−3	−4	64	6	Free-choice paradigm
Self-easy	−3	−11	64	6	Free-choice paradigm
Difference wave	4	−2	61	6	Free-choice paradigm
Incorrect trials	−3	−4	64	6	Flanker task
Correct trials	4	−3	66	6	Flanker task
Difference wave	−3	−4	64	6	Flanker task

### ERP correlates of cognitive dissonance

Initially, we analyzed cognitive dissonance-related activity in frontocentral sites (FCz, Cz, and Fz), which often demonstrate the largest ERN. Indeed, we found that the largest cognitive dissonance-related activity was observed in FCz. Figure 3*B* shows the grand-averaged ERPs and the difference wave at the frontocentral midline electrode FCz, as well as a topographical map of the voltage distribution for the difference wave (Self-difficult − Self-easy trials). A *t* test for the mean ERP amplitude (Self-difficult trials vs Self-easy trials, FCz, time window = 36 ms) revealed a significant effect of cognitive dissonance: *t*_(41)_ = −2.190, *p* = 0.032. The Self-difficult trials (associated with stronger cognitive dissonance) evoked a significantly larger negative frontocentral deflection at a latency of 46 ms than the Self-easy trials.

For a more conservative analysis of the differential ERPs (Self-difficult trials vs Self-easy trials), we applied paired *t* tests with a permutation procedure based on cluster statistics (Maris and Oostenveld, 2007). We found a cluster of electrodes that survived a 200-random-iteration permutation procedure (Fig. 3*B*, magenta crosses). The analysis showed that the cluster of 17 temporal and frontocentral electrodes showed significant difference according to the permutation procedure.

Similar to the Eriksen Flanker task, we applied the LORETA transform to explore the source generator of cognitive dissonance-related activity. The LORETA time window's parameters matched those of the Flanker task (±11 ms around the grand-averaged maximum peak for Self-difficult trials, Self-easy trials, and difference waves). As Figure 3*A* shows, sources of the difference wave were localized in the occipital cortex, ventral prefrontal cortex, and the pMFC, with the greatest activity occurring within the BA6. Overall, the frontocentral distribution of cognitive dissonance-related evoked activity in the free-choice paradigm and its pMFC origin (Fig. 3*B*) were very similar to the frontocentral distribution of ERN in the standard Flanker task (Fig. 3*A*).

We also analyzed ERPs in Computer trials. As mentioned above (see Behavioral correlates of postdecisional preference change), the neural mechanisms underlying participants' responses in Computer trials might reflect complex cognitive processes (e.g., error observation or response inhibition), differentiating this condition from other conditions. Nevertheless, we performed a *t* test of the ERP peak amplitude (Self-difficult trials vs Computer choice trials, FCz) that confirmed a significant effect of cognitive dissonance: *t*_(41)_ = −2.226, *p* = 0.032. As expected, we found no significant difference in the ERP peak amplitude (FCz) between Self-easy trials and Computer trials (*t*_(41)_ = −0.452, *p* = 0.654) and a significant difference between Self-difficult trials and Self-easy trials (*t*_(41)_ = −2.190, *p* = 0.034).

### Relationship between ERP correlates of cognitive dissonance and postdecisional preference changes (SoA)

**Figure 4.**
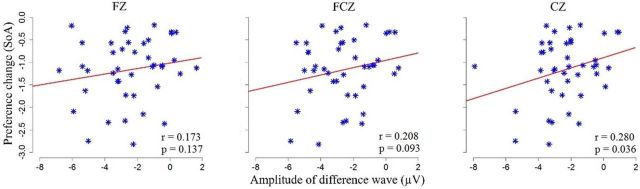
Relationships between cognitive dissonance-related difference wave (Left, Maximum voltage at Fz; Middle, FCz; Right, Cz) and the SoA magnitude for rejected items in Self-difficult trials.

To examine the relationship between the magnitude of the ERN-like correlates of cognitive dissonance and postdecisional preference changes, we investigated the relationship between the aforementioned evoked activity and individual preference changes for rejected items. We calculated the Pearson correlation analysis (Fig. 4) for the difference waves (Self-difficult trials − Self-easy trials at three midline electrodes: FCz, Fz, and Cz) and SoA effects for rejected items (in Self-difficult trials): We found a trend of significant positive correlation at both the FCz (*r* = 0.208, *p* = 0.093) and Cz (*r* = 0.280, *p* = 0.036) electrodes. Thus, the stronger ERN-like correlates of cognitive dissonance were observed (Choice task), and the stronger individual preferences were later changed for rejected items (Preference task II).

### Resting-state neuronal dynamics predict postdecisional preference changes (SoA), and the amplitude of the ERP correlates of cognitive dissonance

We correlated the individual magnitude of SoA with the LRTC scaling exponents that described dynamics of alpha oscillations recorded during rest at the beginning of the experiment. We found a significant negative correlation (*r* = −0.38, *p* = 0.029) between LRTC scaling exponents (8–13 Hz) at the frontal ROI (see Materials and Methods) and the SoA for rejected items in Self-difficult trials. This correlation indicates that the more pronounced frontal LRTC at the rest predicts a stronger decrease in preference for the rejected items in Self-difficult trials later on.

**Figure 5.**
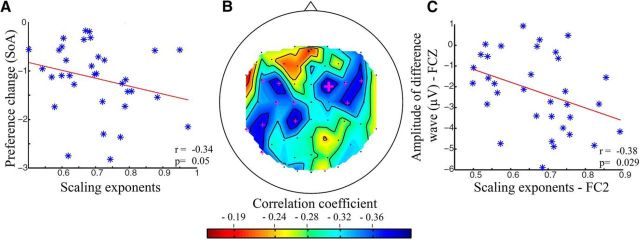
***A***, A relationship between LRTC scaling exponents and SoA magnitude for the rejected items in Self-difficult trials. ***B***, Topography of the correlation between LRTC scaling exponents and the amplitude of the difference wave (Self-easy − Self-difficult trials). Cross represents electrodes belonging to a significant cluster. ***C***, A scatter-plot showing a relation between the LRTC scaling exponents (FC2 electrode) and the amplitude of the difference wave (Self-difficult − Self-easy trials) at FCz electrode. Red line indicates the linear least-squares fit.

Because evoked responses at the FCz electrode showed the most significant difference between Self-easy and Self-difficult trials, we correlated amplitude measures from this electrode with LRTC scaling exponents and the amplitude of alpha oscillations recorded at rest. Our analysis showed that LRTC scaling components (8–10 Hz band) were correlated negatively (*p* < 0.05) with the cognitive dissonance-related difference wave (Self-difficult trials − Self-easy trials). Figure 5*B* shows a topography of this correlation. The significant cluster is widely distributed, covering the frontal and central part of the head. The amplitude of the alpha oscillations recorded during rest did not correlate with the evoked responses (Fig. 5*C*). Overall, our findings indicate that a more pronounced LRTC leads to larger ERP and behavioral correlates of postdecisional adjustments of preferences.

## Discussion

Similar to previous studies (Brehm, 1956; Kitayama et al., 2004; van Veen et al., 2009; Izuma et al., 2010; Mengarelli et al., 2015), our behavioral results demonstrate that decisions induce preference changes: Individuals were more likely to downgrade their preferences for rejected items to align them with their actual choices.

A previous neuroimaging study (Izuma et al., 2010) showed the neural signature of choice-induced preference change during the rerating of options, the paradigm which was also used in our study. The authors found that the pMFC activity reflected the degree of cognitive dissonance and predicted the strength of choice-induced preference changes. Moreover, a recent transcranial magnetic stimulation study demonstrated a causal role for the pMFC in choice-induced preference change: Repetitive transcranial magnetic stimulation of the pMFC following the choice stage significantly reduced choice-induced preference changes compared with control stimulations over a different brain region (Izuma et al., 2015). Therefore, Izuma (2013) concluded that the activity of the pMFC reflects internal consistency between one's opinions and behaviors and associated cognitive dissonance with the processes underlying changes in opinions and behaviors.

Although cognitive dissonance has traditionally been investigated using the free-choice paradigm (Brehm, 1956; Harmon-Jones and Harmon-Jones, 2007), little is known about the cognitive and neural processes that occur during the decisional stage (as an exception, see Jarcho et al., 2011) or their role in follow-up postdecisional preference changes.

To the best of our knowledge, the present study is the first EEG study to directly investigate neural correlates of cognitive dissonance during the decisional process. Our ERP data suggest that choices associated with stronger cognitive dissonance trigger a greater negative frontocentral ERN-like deflection with the maximum at 60 ms (after the choice). The location, source localization, and timing (Fig. 3*B*) of the negative frontocentral deflection in Self-difficult trials closely resemble ERN (for a review of ERN′s characteristics, see Gehring et al., 2011). Nevertheless, further studies are needed to confirm the identity of choice-related activity during the free-choice paradigm with ERN (Miltner et al., 1997; Gehring and Willoughby, 2002; Holroyd and Coles, 2002; Nieuwenhuis et al., 2004; Nieuwenhuis et al., 2007). Furthermore, amplitudes of ERN-like potentials predicted individual differences in postdecisional preference changes: A larger ERN-like potential was associated with larger preference changes.

In the control study, we recorded the standard ERN during the Flanker task (Falkenstein et al., 1995; Holroyd and Coles, 2002; Gehring et al., 2011) to test the spatial and temporal correspondence of the ERN-like potential generated during the free-choice paradigm with standard ERN. Our results show a strong similarity between the spatial and temporal characteristics of both evoked responses. A difference in the amplitudes of the Eriksen Flanker task and ERN-like potential in the free-choice paradigm may be due to difference in task difficulty. Because the Eriksen Flanker task is simpler than the free-choice paradigm, a smaller ERN-like potential could reflect more complex and slower mechanisms underlying relatively complex decisions during food choices. Previous studies demonstrated that ERN is indeed susceptible to changes in error salience or attention (Hajcak et al., 2005; Riesel et al., 2012).

The recent transcranial magnetic stimulation study demonstrated a causal role of the pMFC in postdecisional preference changes (Izuma et al., 2015). Off-line downregulation of the right pMFC just after the Choice task induced a reduction of choice-induced preference changes (SoA). Importantly, our ERP study suggests that an earlier neural process might also be involved in the subsequent preference changes for rejected items. Our results indicate that, during choices associated with strong cognitive dissonance, the pMFC is already generating a neural error-signal reflecting the need for behavioral adjustments similar to ERN.

Furthermore, an important role of the pMFC in cognitive dissonance and choice-induced preference changes (Izuma et al., 2010) suggests that its ongoing, spontaneous (resting state) activity may affect follow-up neural and behavioral effects of cognitive dissonance. For the quantification of spontaneous oscillatory dynamics, we used LRTC, which captures neuronal activity at different time scales. The presence of LRTC is consistent with the idea that neuronal networks might operate at a critical state (Linkenkaer-Hansen et al., 2001; Poil et al., 2012) that could be beneficial for the optimal processing of information in the brain (Shew and Plenz, 2013). For example, recent studies have shown that the perception of near-threshold sensory stimuli (Palva et al., 2013) and the precision in sensorimotor tasks (Smit et al., 2013; Samek et al., 2016) are related to the LRTC of neuronal oscillations recorded at rest. We went a step further and demonstrated that LRTCs at rest are also associated with more complex cognitive processes, such as cognitive dissonance-induced preference changes, or SoA effects. Thus, the SoA-related reconfiguration of neuronal value representations might require efficient rerouting of synaptic inputs and their consecutive stabilization: processes best implemented by the delicate balance of excitation and inhibition within specialized neural microcircuitry (Rolls et al., 2008).

Importantly, LRTCs are indeed most pronounced when excitation and inhibition are balanced (Poil et al., 2012). Thus, individuals with stronger LRTC at frontal cortices might demonstrate a larger SoA, which was indeed observed in the present study. Interestingly, we also found that LRTC in frontal areas predicted not only behavioral outcomes of the follow-up free-choice paradigm but were also correlated with the evoked brain responses when choosing between two items. This finding is in line with the general conception that LRTCs of alpha oscillations, recorded at rest, are likely to reflect large-scale cortical excitability (Fedele et al., 2016) and could therefore also be related to the generation of ERP.

Together, our electrophysiological results suggest that pMFC activity might play a critical role in modulating postdecisional preference changes occurring when difficult decisions between similarly attractive options are faced. Although previous studies found similar activity in the late stages of the decisional process in the free-choice paradigm (Preference task II), our data favor a central role of the pMFC in cognitive dissonance detection during the decisional process (Choice task). Indeed, our study demonstrates that difficult decisions (high cognitive dissonance) trigger a more prominent ERN-like neural signal than easy decisions. ERN has been thought to reflect error detection (Luu et al., 2004), conflict detection, conflict monitoring (Yeung et al., 2004), and cognitive control (Ridderinkhof et al., 2004), as well as serving as an important electrophysiological correlate of reinforcement-learning mechanisms (Holroyd and Coles, 2002). The existence of ERN has been proven in a large set of experimental designs and paradigms, such as the Stroop task and Flanker task. In our experiment, we found similar frontocentral activity during the free-choice paradigm and Flanker task. Thus, our ERP data support the hypotheses that (1) pMFC activity during the decisional process plays a key role in preference modulation; and (2) neural mechanisms of choice-induced preference changes might be similar to more general reinforcement-learning mechanisms.

Interestingly, some studies have shown that the left dorsolateral prefrontal cortex (DLPFC) is also involved in cognitive dissonance (Harmon-Jones and Harmon-Jones, 2008; Harmon-Jones et al., 2011; Mengarelli et al., 2015). As the DLPFC has been shown to be implicated in cognitive control (Miller and Cohen, 2001), it was suggested that the DLPFC is not directly involved in cognitive inconsistency reduction; rather, its activity is believed to be related to a more general cognitive control process (Izuma et al., 2015) and the implementation of performance adjustment (Ridderinkhof et al., 2004). As in Ridderinkhof et al. (2004), the DLPFC and pMFC might have a functional interaction that permits the monitoring and execution of performance adjustment. Further studies are clearly needed to fully uncover how the activity of the pMFC modulates subsequent preference changes as well as the role of the pMFC-DLPFC network in cognitive dissonance reduction. The present study also does not allow for the investigation of the relationship between the amplitude of electrophysiological signatures of difficult choices and the level of postdecisional adjustments on a trial-by-trail basis. A new specially designed version of the free-choice paradigm could allow for the study of this relationship in the future. In conclusion, our results provide strong evidence that postdecisional preference changes and performance monitoring demonstrate similar neural signatures. Choice-induced preference changes are reflected in the choice-related activity of the pMFC as part of the general performance-monitoring circuitry. Furthermore, resting-state dynamics determine both behavioral and neural correlates of postdecisional preference changes. Thus, neurocomputational mechanisms of choice- and feedback-induced preference changes may be more strongly intertwined than previously thought.
